# Complete Genome Sequence of Human Coronavirus NL63 CN0601/14, First Isolated in South Korea

**DOI:** 10.1128/genomeA.00152-18

**Published:** 2018-03-22

**Authors:** Jeong-Min Kim, Seung-Tae Kim, Jeong-Sun Yang, Sung Soon Kim, Hyang-Min Cheong

**Affiliations:** a Division of Emerging Infectious Disease and Vector Research, Center for Infectious Diseases Research, Korea National Institute of Health, Korea Centers for Disease Control and Prevention, Cheongju-si, South Korea; b Division of Vaccine Research, Center for Infectious Diseases Research, Korea National Institute of Health, Korea Centers for Disease Control and Prevention, Cheongju-si, South Korea

## Abstract

We report here the complete genome sequence of the human coronavirus NL63 CN0601/14 strain, first isolated from South Korea. It contains 18-nucleotide discontinuous deletions of the open reading frame 1a (ORF1a) and spike regions. This study will aid in our understanding of the complete genome sequences of isolated coronaviruses in South Korea.

## GENOME ANNOUNCEMENT

Human coronavirus (HCoV) NL63 is a member of the family Coronaviridae in the order Nidovirales (1, 2). The genome is a large-envelope virus with a positive-sense single-stranded RNA genome of approximately 28 kb in length and contains a cap structure at its 5ʹ end and a poly(A) tail at its 3ʹ end. The genome consists of 5ʹ and 3ʹ noncoding regions (NCRs), the 1a and 1b genes that encode the nonstructural polyproteins divided by host and viral proteases, and the genes encoding four structural proteins, spike (S), envelope (E), membrane (M), and nucleocapsid (N). Furthermore, the open reading frame 3 (ORF3) gene is located between S and E, which all synthesize a nested set of multiple subgenomic mRNAs (3, 4). HCoV NL63 was discovered in the Netherlands in 2004 (2), has spread worldwide, and is detected frequently in the winter (5, 6). The virus is associated with acute respiratory tract infections, such as croup, bronchiolitis, and pneumonia, in young children and the elderly (7–11). In South Korea, since HCoV NL63 was first observed in November 2004, it has also been detected frequently in the winter. Here, we announce the complete genome sequence of the HCoV NL63 CN0601/14 strain, first isolated in South Korea.

The strain CN0601/14 was isolated from a patient infected with HCoV NL63 in 2014. Total RNA was extracted from the infected cell supernatant using the QIAamp viral RNA minikit (Qiagen, Germany), according to the manufacturer’s instructions. The complete genome of CN0601/14 was generated by first-strand cDNA synthesis from the total RNA (300 ng) with primers consisting of oligo(dT) and 3ʹ-untranslated region (UTR) reverse primer and performed with PCR using 20 pairs of primers amplifying 10 overlapped fragments. The primers were designed using CLC Main Workbench version 7.0.3 (Qiagen) based on the sequence of the HCoV NL63 isolate CBJ 037 genome (GenBank accession number JX104161). The 10 amplified PCR products were purified and sent to GnC Bio Co. for sequencing. The complete genome of CN0601/14 (27,535 nucleotides [nt]) compared to that of the prototype HCoV NL63 virus (accession number NC005831) by multiple alignment showed that there are 18-nt discontinuous deletions, with a 15-nt deletion in positions 3035 to 3049 of the ORF1a region and a 3-nt deletion in positions 327 to 329 of the spike region. Furthermore, the genome of CN0601/14 showed 98.76% nucleotide homology with that of the prototype HCoV NL63 virus.

Altogether, the complete genome sequence data of CN0601/14 indicate that variations in HCoV NL63 occur in South Korea. This information will aid in the understanding of the genetic diversity of HCoV NL63 in South Korea and in manufacturing an infectious CN0601/14 clone construct based on Korean vaccine candidate development.

### Accession number(s).

The complete genome sequence of the human coronavirus NL63 CN0601/14 strain is available in GenBank under the accession number MG772808.
